# Progress in Microbial Degradation of Feather Waste

**DOI:** 10.3389/fmicb.2019.02717

**Published:** 2019-12-05

**Authors:** Qingxin Li

**Affiliations:** Guangdong Bioengineering Institute (Guangzhou Sugarcane Industry Research Institute), Guangdong Academy of Sciences, Guangzhou, China

**Keywords:** keratin, microorganism, protease, keratinase, bio fertilizer, fermentation

## Abstract

Feathers are a major by-product of the poultry industry. They are mainly composed of keratins which have wide applications in different fields. Due to the increasing production of feathers from poultry industries, the untreated feathers could become pollutants because of their resistance to protease degradation. Feathers are rich in amino acids, which makes them a valuable source for fertilizer and animal feeds. Numerous bacteria and fungi exhibited capabilities to degrade chicken feathers by secreting enzymes such as keratinases, and accumulated evidence shows that feather-containing wastes can be converted into value-added products. This review summarizes recent progress in microbial degradation of feathers, structures of keratinases, feather application, and microorganisms that are able to secrete keratinase. In addition, the enzymes critical for keratin degradation and their mechanism of action are discussed. We also proposed the strategy that can be utilized for feather degradation. Based on the accumulated studies, microbial degradation of feathers has great potential to convert them into various products such as biofertilizer and animal feeds.

## Introduction

**FIGURE 1:**
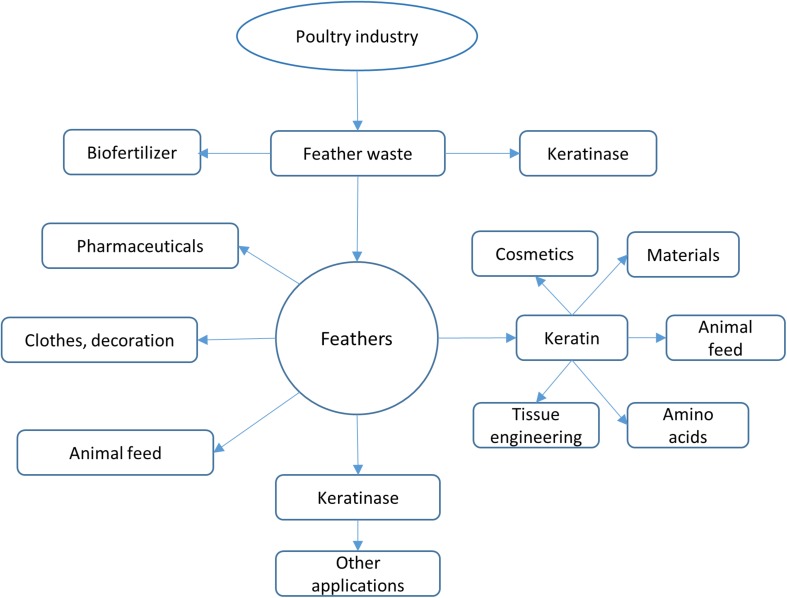
Feather applications. Feathers from poultry industry can be converted into various products. Feathers can be used directly or processed using different ways such as chemical treatment and microbial processing. The most attractive strategy to use feathers is to convert them into value-added products such as biofertilizer and high nutrient animal feeds.

Feathers are an important by-product in poultry industry as they account for 5–7% of the body weight of chicken. It is estimated that approximately several million tons of feathers could be generated annually from poultry industry globally (Verma et al., 2017; da Silva, 2018). Feathers are usually collected and stored at certain areas before further treatment. As feathers might be mixed with blood, meat and grease, the storage conditions such as temperature and duration have to be carefully managed (Tesfaye et al., 2017). Feathers can be disposed by incineration, which is one effective method to destroy conceivable infection agents. Feathers can also be disposed through burial and controlled landfilling while special management is required to keep them from ground water (Tesfaye et al., 2017). Feathers have many applications. As described in a recent review (Tesfaye et al., 2017), feathers such as chicken feathers can be used for decorative applications, medical devices, fertilizer, dusters, bedding materials, and feedstocks (Figure 1) (Papadopoulos et al., 1986). The traditional feather processing methods such as chemical treatment and stem pressure cooking method could convert feathers into animal feeds, but the processes need a large amount of energy and some amino acids are destroyed during treatment (Papadopoulos, 1989; Latshaw et al., 1994; Wang and Parsons, 1997). Although feathers can be utilized as materials in different fields (Shanmugasundaram et al., 2018), a large amount of feathers are still released into the environment without proper treatment. Feathers have become one source of pollutant because of their recalcitrant nature (Brandelli et al., 2015). Untreated feather waste can sustain many pathogenic microorganisms and emit various pollutants such as nitrous oxide, ammonia, and hydrogen sulfide, which are a threat to the environment and people’s health (Tamreihao et al., 2019). Therefore, converting feathers into value-added products using economic methods is of great interest to many researchers (Kang et al., 2018). Accumulated studies have shown that feathers can be efficiently degraded by various microorganisms (Figure 1). Microbial conversion of feathers into value-added products such as biofertilizer and animal feeds should be used in poultry industries (Tamreihao et al., 2019).

In this review, progress in microbial degradation of feathers is described. Some bacteria and fungi used in feather degradation are summarized. As most studies used only a single strain in feather treatment, using microbial consortium in the treatment might be a more effective way in feather degradation. The enzymes that are secreted by microorganisms and their mechanism of action on protein degradation are discussed. Based on accumulated studies, feathers from poultry industry can be treated in an economic and environmentally friendly way and serve as a valuable source for other applications.

## Keratin and Its Structures

The main component (over 90%) of feathers is keratin which is also a key structural element in other organs. Other components such as keratin-associated proteins are present in both feathers and other keratin-rich materials (Adav et al., 2018). Keratin forms fibrous structures and exists widely in nature. It is a recalcitrant structural protein and the third most abundant material after cellulose and chitin (Lange et al., 2016). Keratin can be divided into soft keratin whose cysteine content is less than 10% and hard keratin whose cysteine content is 10–14% (Jin et al., 2017). Hard keratin is normally present in hair, nails, wool, claws, and hooves while soft keratin is present in the epidermis of skin protecting cells from stress. Keratin forms recalcitrant polymers which are insoluble in water and organic solvent, resistant to degradation by the common proteolytic enzymes such as pepsin and trypsin. This is due to the fact that keratin forms high order fibrous structures through disulfide bonds, hydrogen bonds and hydrophobic interactions (Meyers et al., 2008).

Like normal proteins, keratins’ secondary structures contain both α-helix and β-sheet. Keratins can be classified as α-keratins and β-keratins, respectively. β-keratin contains a central domain which is rich in residues favoring to form β-sheet structures associated with the filament framework, an N- and a C-terminal domains which are associated with the matrix and form crosslinking via disulfide bonds (Fraser and Parry, 2011). The structures of β-pleated sheets are present in β-keratins and can form supramolecular fibril bundle. The α-helix coiled and coil structures are assembled in α-keratins and can form intermediate filaments through self-assembling (Lange et al., 2016). The contents of α- and β-keratins are different among various organs (Wu et al., 2015). For example, wool is normally composed of α-keratin (Fang et al., 2013) while feathers contain both keratins (Bodde et al., 2011). Based on analysis, the contents of α- and β-keratins at various position of feathers are different (Ng et al., 2014). The outer rachis of feathers consists mainly β-keratin. As β-keratins have a higher content of cysteine residues than α-keratins, a higher content of disulfide bonds is present (Tesfaye et al., 2017). The formation of covalent bonds is able to stabilize protein structure, which makes it difficult for feathers to be degraded by proteases (Wang et al., 2016).

Posttranslational modifications on keratins such as phosphorylation, sumoylation and glycosylation might also be important for their structure and stability (Snider and Omary, 2014). In some keratins, the uncoiled head structure contains both threonine and serine amino acids which could be the phosphorylation sites. Site-specific phosphorylation of the residues located at this domain could be a major facilitator of intermediate filament reorganization (Snider and Omary, 2014). As keratins can be classified into type I (acidic) and type II (basic) keratins based on the pH at which the proteins are neutral with total charges equal to zero (PI) (Bragulla and Homberger, 2009). Post-translational modification can affect the PI of keratins. Therefore, the conformation of keratins will be changed when the post-translational modification status is altered. If the modification site is mutated, lacking post-translational modification may lead to unsuccessful intermediate filament assembly (Bragulla and Homberger, 2009).

## Feather Applications and Process

Keratin can form high-molecular weight complex without chemical modifications, making it attractive to be used as novel materials in many fields (Barone et al., 2005). Feathers from poultry have great potential to be used in several fields as they are one of the important source of keratin (Sharma and Gupta, 2016). As keratins can also be obtained from human resources, their corresponding products can be used as biomedical devices (Dias et al., 2010a). It was shown that keratin material derived from human hair clippings could be used as a new carrier/scaffold for carrying bone morphogenetic protein-2 in bone regeneration (de Guzman et al., 2013). Keratins were considered as a biodegradable material and have been used in wound healing applications (Wang et al., 2017). Keratins from different sources were proven to have biocompatibility with living systems, which makes them have potential applications as novel and valuable materials (Dias et al., 2010b; Ma et al., 2016). Keratins from feathers or other wastes can be converted into amino acids (Hill et al., 2010) which can be used as animal feeds (Onifade et al., 1998). Feathers from chickens were able to be converted into hydrogels for wound healing in a rat model (Wang et al., 2017). In addition, feathers also contain fat, water and minerals such as nitrogen, phosphorus, potassium, calcium, magnesium, iron, manganese, zinc, and copper, which makes them to serve as valuable energy and material sources (Tamreihao et al., 2019). Feathers of course can serve as an important source of amino acids as the keratin sequence consists of high contents of cysteine, glutamine, proline, and serine amino acids (Tesfaye et al., 2017). The contents of histidine, glycine, tryptophan, and glutamic acid are low. The feather hydrolysis products can serve as protein or amino feeds for animals or for microbial growth (Williams et al., 1991). Therefore, feathers can serve as important carbon and nitrogen sources for microbial culture.

Despite the potential applications of keratins derived from keratin-rich materials, feathers from poultry industries are not used efficiently. The feather by-products are normally mixed with other wastes such as blood, meat, skin, fat, and poultry dung (Tesfaye et al., 2017). Although feathers can be converted into feather meal by steam and chemical treatment (Kim and Patterson, 2000), it requires complicated steps in the poultry industry. Feather-containing wastes from poultry industry have to be stored properly before different treatment (Tesfaye et al., 2017). Storage of feather wastes is not a straightforward process as several parameters such as space, temperature and time need to be properly managed. Improper storage can cause growth of bacteria, yeast, fungi, and viruses or the formation of odors such as ammonia and hydrogen sulfide, which can threaten people’s health (Tesfaye et al., 2017). If feather wastes contain other impurities, separation is required before they are sent to process. Therefore, microbial processing of feathers from poultry industries is an economic and efficient way as feather wastes can be processed directly without pretreatment such as separation.

## Microbial Degradation of Feathers

Although the nature of keratin-rich wastes such as feathers is resistant to degradation by common proteases, keratins are not accumulated in nature, suggesting that they are degraded by microorganisms (Williams and Shih, 1989). Studies have shown that many microorganisms are able to degrade such wastes by secreting keratinolytic and proteolytic enzymes-keratinases (Williams and Shih, 1989; Tamreihao et al., 2019). These microorganisms include bacteria, actinomycetes, and fungi (Cǎlin et al., 2017; Bohacz and Korniłłowicz-Kowalska, 2019). Numerous microorganisms have been isolated from different environments that are rich in keratin and have been applied to degrade keratin-containing wastes from different resources (Onifade et al., 1998; Chaturvedi et al., 2014).

### Bacteria

Quite a few bacteria have been isolated from various environments especially the places rich in keratin-containing materials (Muthusamy et al., 2011). Soil samples from the poultry industry are a good source for isolating feather-degrading bacteria (Kim et al., 2001). The most frequently used method to identify screened strains is 16S rDNA sequencing (Khodayari and Kafilzadeh, 2018). The isolated bacteria were then used in degrading keratin-rich materials such as feathers and wools. Secreted proteases-keratinase by these organisms were responsible for cleaving the keratin proteins. Recent studies suggested that keratinases were functional synergistically with other enzymes. Bacteria have great potential to be used widely as they grow fast and their enzymes sustain the activity under different conditions. Some enzymes are still active at high temperature and at different pHs. *Bacillus licheniformis* is the most effective keratin-degrading bacterium in the genus (Manczinger et al., 2003). Other bacteria including *Bacillus*, *Stenotrophomonas*, *Pseudomonas*, *Brevibacillus*, *Fusarium*, *Geobacillus*, *Chryseobacterium*, *Xanthomonas*, *Nesterenkonia*, and *Serratia* are able to produce keratin-degrading enzymes (Table 1). The keratinases produced by bacteria exhibited a wide range of optimal temperature (28–90°C) and pH (5.8–11) (Tamreihao et al., 2019). The molecular weights of bacterial keratinase are different among different species. In addition, adding keratin or feathers in the cultural medium normally facilitates production of keratinase (Herzog et al., 2016), implying that the enzyme production might be an inducible process.

**TABLE 1:** Some bacteria having capability to degrade feathers.

**Strain**	**Remarks**	**References**
*Bacillus amyloliquefaciens*	Two extracellular keratinolytic proteases produced by S13 were purified. These two enzymes were with 47 and 28 kDa, respectively	Hamiche et al., 2019
*Bacillus cereus*	A strain KB043 was shown to be able to produce keratinase	Swetlana and Jain, 2010
	This strain producing keratinase was screened from the halophilic environment	Arokiyaraj et al., 2019
	A strain was able to degrade feathers by producing keratinase	Jeevana Lakshmi et al., 2013
	A strain Wu2 was able to produce keratinolytic enzyme using feather as the sole carbon and nitrogen sources	Lo et al., 2012
*Bacillus thuringiensis*	This stain is able to degrade heat-treated feather. Additives in the medium affected feather degradation	Sahni et al., 2015
	A strain AD-12 was able to produce detergent-stable serine keratinolytic proteinase with a molecular weight of 39 kDa	Gegeckas et al., 2014
*Stenotrophomonas maltophilia*	This stain was isolated from the gut of a spider. Three enzymes were purified from this strain	Saravanan and Dhurai, 2012
	Strain BBE11-1 secrets two keratinolytic proteases. These two enzymes-KerSMD (48 kDa) and KerSMDF (40 kDa) were overexpressed in *E. coli*	Fang et al., 2014
	A strain R13 was able to produce keratinolytic enzyme using chicken feathers as the sole carbon and nitrogen sources	Jeong et al., 2010b
	A strain BBE11-1 was able to secrete keratinase and degrade wool waste	Fang et al., 2013
	A strain R13 was isolated and able to produce keratinolytic enzyme in the chicken feather medium	Jeong et al., 2010b
*Bacillus* sp.	Bacillus strains were able to produce keratinase	Lin et al., 1999; Gegeckas et al., 2018
	Three strains were used to convert feather into feather hydrolysate	Callegaro et al., 2018
	Quite a few *Bacillus* sp. strains were screened from marine environment and produced keratinase	Herzog et al., 2016
	A strain was able to degrade feather by producing alkaline keratinase and disulfide reductase	Rahayu et al., 2012
	A metalloprotease with a molecular weight of 134 kDa was purified from the strain	Lee et al., 2002
*Bacillus aerius* NSMk2	Complete degradation of white chicken feather was observed in 3 days	Bhari et al., 2018
*Bacillus thuringiensis*	This stain is able to degrade heat-treated feather. Additives in the medium affected feather degradation	Sahni et al., 2015
	A strain AD-12 was able to produce detergent-stable serine keratinolytic proteinase with a molecular weight of 39 kDa. The enzyme was characterized	Gegeckas et al., 2014
*Bacillus licheniformis*	A strain K-508 was isolated having feather degrading activity and its fermentation product exhibited protease activity	Manczinger et al., 2003
	A gene of keratinolytic protease was identified in strain PWD-1 that could produce keratinase	Lin et al., 1992, 1995
	A strain K-508 was able to degrade feather with several proteases secreted	Manczinger et al., 2003
	The crude enzyme produced by strain ALW1 was able to degrade native feather up to 63% in redox free system	Abdel-Fattah et al., 2018
	The strain ATCC 21415 was used to treat biostimulants which can affect bioremediation of soil	Rodríguez-Morgado et al., 2015
	Extracellular proteins of this strain were identified when the strain used different feathers as substrates	Parrado et al., 2014
	The keratinase from strain BBE11-1 was mutated based on computational design. The mutant was expressed in *B. subtilis* and exhibited enhanced thermal stability	Liu et al., 2013
	A strain ER-15 was able to produce a 58 kDa keratinase which could hydrolyze several protein complexes	Tiwary and Gupta, 2010
	The keratinase produced in this stain was expressed in *B. subtilis*	Wang and Shih, 1999
*Bacillus subtilis*	Whole cell mutagenesis was used to improve the enzymatic activity	de Paiva et al., 2018
	A strain DP1 was isolated and was able to produce keratinase that was stable range of pH (8–12) and temperature (20–50°C)	Sanghvi et al., 2016
	A strain PF1 was used to simultaneously produce keratinolytic protease and other enzymes using feather containing medium	Bhange et al., 2016b
	A strain NRC3 was able to produce thermal stable metallo-keratinase (32 kDa)	Tork et al., 2013
	A strain BF11 was able degrade feather	Jeevana Lakshmi et al., 2013
	A strain RM-01 produced keratinase in solid-state fermentation using chicken feathers as substrate	Rai et al., 2009
	Strain S8 was able to degrade feather and produce indoleacetic acid. This strain also exhibited antifungal activities	Jeong et al., 2010a
	A strain was able to degrade feathers and the products could also inhibit bacterial growth	Liu et al., 2017
*Bacillus pumilus*	Adding cysteine in feather medium could increase enzyme activity	Kim et al., 2005
	A strain was able to produce keratinase using feather as substrate. The produced enzyme was able to remove the blood stains from cloth without affecting its fiber properties	Ramakrishna Reddy et al., 2017
	A strain FH9 was able to produce keratinase which was characterized. KS12 produced a thermal stable enzyme	Rajput et al., 2010; Abdel-Naby et al., 2017
	A strain A1 was able to degrade feathers and the produced feather protein hydrolysate exhibited antioxidant activity	Fakhfakh et al., 2011
*Bacillus sphaericus* and *Bacillus thuringiensis israelensis*	This entomopathogenic bacterial were able to degrade feathers, indicating that this waste can be converted into mosquitocidal biopesticides	Poopathi and Abidha, 2008
*Bacillus tequilensis*	A 28 kDa protease was overexpressed in *E. coli* and purified	Zaraî Jaouadi et al., 2015
*Bacillus pseudofirmus*	A strain FA30-1 was able to degrade feather completely and an enzyme was purified	Kojima et al., 2006
*Brevibacillus parabrevis*	A surfactant-resistant enzyme was purified from this strain	Zhang et al., 2016
*Bacillus megaterium*	A strain SN1 was able to degrade feather and produce caesinolytic enzyme in feather medium	Agrahari and Wadhwa, 2012
	A strain was found to be able to degrade feathers and other keratin-rich materials	Park and Son, 2009
*Brevibacillus* sp.	Production and purification of one 83.2 kDa keratinase from strain AS-S10-II were carried out	Rai and Mukherjee, 2011
*Chryseobacterium sediminis*	This strain was able to grow using feather as sole carbon and nitrogen sources. It degraded feather and antioxidant and indole-3-acetic acid production were observed	Kshetri et al., 2019
*Fusarium* sp.	This strain was found to be efficient in keratin degradation	Cǎlin et al., 2017
*Fervidobacterium islandicum*	A thermophilic anaerobe was able to produce amino acids by degrading feathers. A 97 kDa enzyme could form oligomers	Nam et al., 2002
*Stenotrophomonas* sp.	Screened strains mostly identified as *S. maltophilia* and *S. rhizophila* were able to produce keratinase	Herzog et al., 2016
*Geobacillus stearothermophilus*	The genome of this strain encodes a keratinolytic protease which was overexpressed in *E. coli*. The enzyme was purified and characterized	Gegeckas et al., 2015
*Chryseobacterium* sp.	A bacterium kr6 was able to produce feather hydrolysates which exhibited antioxidant and antihypertensive activities	Lin et al., 1992; Fontoura et al., 2014
	Effect of nutritional conditions on enzyme product by kr6 was explored	Riffel et al., 2011
*Micrococcus* sp.	A study showed that this strain was able to produce several keratinases with high molecular weights	Laba et al., 2015
*Pseudomonas stutzeri*	This strain K4 was able to metabolize chicken feather. It could produce five keratinases	Chaturvedi et al., 2014
*Pseudomonas aeruginosa*	A 33 kDa keratinase was purified from strain C11 which could degrade feathers	Han et al., 2012
*Pseudomonas* sp.	A 30 kDa keratinase was isolated from a Pseudomonas strain	Tork et al., 2010
*Paenibacillus woosongensis*	A strain could grow in a feather medium and produce keratinases. The resulting product promoted plant growth	Paul et al., 2013
*Xanthomonas* sp.	A strain P5 was able to degrade feather through enzymes	Jeong et al., 2010c
*Nesterenkonia* sp.	A strain AL20 produced protease in the presence of chicken feather. The substrate specificity was explored	Bakhtiar et al., 2005
*Serratia* sp.	A feather hydrolyzing enzyme was obtained from this strain. Feather substrate was able to increase the enzyme production. This enzyme was active at 60°C and pH 10	Khardenavis et al., 2009
*Stenotrophomonas* sp.	A strain D-1 was isolated and able to degrade chicken feather at 20°C in 2.5 days	Yamamura et al., 2002a
*Serratia marcescens*	Strain P3 was able to be produce a 53 kDa keratinase belonging to the serralysin family	Bach et al., 2012
*Vibrio* sp.	A strain was able to degrade feathers	Bockle and Muller, 1997; Grazziotin et al., 2007

Extensive studies have been carried to isolate different types of keratin-degrading bacteria. Random mutagenesis using ethyl methanesulfonate was carried out to improve the activity of a keratin-degrading bacterium *Bacillus subtilis* LFB-FIOCRUZ 1266. The mutants exhibited higher feather degradation rate by 15% than the wild type strain. In addition, the mutants showed higher keratinolytic activity and sulfide yield than the wild type strain (de Paiva et al., 2018). Mutation using ultraviolet irradiation and N-methyl-N′-nitro-N-nitrosoguanidine treatment or N-methyl-N′-nitro-N-nitrosoguanidine treatment alone was carried out on *B. subtilis*. The resulting mutant exhibited higher keratinase activity (Cai et al., 2008) or higher feather degradation efficiency than the wild type. The authors also identified a 45 kDa protease playing important role in feather degradation (Wang et al., 2015). Apparently, random mutagenesis on selected strain is an efficient way to improve feather degradation efficiency, implying multiple enzymes might be involved in the degradation process. Site-directed mutagenesis to improve keratinase activity could also improve feather degradation efficiency (Liu et al., 2013).

### Fungi and Actinomycetes

Degradation of keratin-rich material in nature is the result of cooperation of bacteria and other microorganisms (Lange et al., 2016). Fungi and actinomycetes have been found to be able to degrade keratin-rich materials such as feathers. Some fungi are pathogenic and present on the surface of human or animal skins. The keratinases secreted by these fungi are important for their invasion into the body (Gilardi, 1965). These fungi have special structures such as hyphae to facilitate keratin degradation (Korniłłowicz-Kowalska and Bohacz, 2011; Tridico et al., 2014). It was shown that high keratinase activity correlated with fast development of symptoms (Viani et al., 2001). The pathogenic fungi could secret keratinases while they must be avoided in applications due to the safety requirement. Some non-pathogenic fungi exhibited capability to degrade feathers and have potential for being approved for application in animal feed or biofertilizer (Bhange et al., 2016a). The amino acid sequences of keratinases produced by fungi and actinomycetes are different from those produced by bacteria. The keratinase producing fungi have been reviewed recently (Lange et al., 2016) and Table 2 also lists some fungi and actinomycetes that are able to produce keratinases and have potential to be used in feather degradation. In addition to feather degradation, some strains were able to produce antibiotics which inhibited bacterial growth, which has been demonstrated in several actinomycetes (Pettett and Kurtböke, 2004). This type of strain is very useful in feather treatment as feathers from poultry industries may carry different types of pathogens.

**TABLE 2:** Some strains exhibited keratinase activities and are able to degrade feathers.

**Strain**	**Remarks**	**References**
*Trichoderma harzianum*	A medium containing feather waste was used for enzyme production by strain HZN12	Bagewadi et al., 2018
*Trichoderma atroviride*	A 21 kDa keratinase was obtained from F6 strain	Cao et al., 2008
*Meiothermus taiwanensis*	The strain WR-220 produced a heat stable enzyme with structure determined. The presence of disulfide bonds might be responsible for the high stability	Wu et al., 2017
*Fusarium* sp.	This strain was found to be efficient in keratin degradation	Cǎlin et al., 2017
*Aspergillus niger*	Mutants originated from the strain exhibited different keratinase activities	Mazotto et al., 2013
*Aspergillus parasiticus*	A 36 kDa keratinolytic protease was purified from this strain	Anitha and Palanivelu, 2013
*Purpureocillium lilacinum*	A 37.0 kDa keratinolytic serine protease was produced by this strain. The enzyme was stable in the presence of organic solvents and detergents	Cavello et al., 2013
*Scopulariopsis brevicaulis*	A strain was able to produce a 28.5 kDa keratinase	Sharaf and Khalil, 2011
*Streptomyces* sp.	A proteinase was obtained from strain AB1 with a molecular weight of 29.9 kDa	Jaouadi et al., 2010
	Both submerged and solid-state fermentations were used in keratinase production by strain 594	De Azeredo et al., 2006
	Several strains have been shown to be able to degrade feathers. Antibiotic activity was also observed in these strains	Pettett and Kurtböke, 2004
	Fermentation conditions are critical for keratinase production	Tatineni et al., 2007
*Microsporum canis*	The keratinase from this stain was overexpressed and purified from yeast	Descamps et al., 2003
*Microsporum gypseum*	This strain was used to evaluate keratinase activity. Strains isolated from different resources exhibited different expression levels	Weary et al., 1965; Giudice et al., 2012
*Nocardiopsis* sp.	A strain was able to produce a variety of alkaline hydrolytic enzymes which were stable under acetic conditions	Mitsuiki et al., 2002
*Streptomyces fradiae*	A strain was able to produce a 24 kDa keratinase which was active at alkaline pH and also cleaves ester and amide bonds formed by the residues of aromatic and basic amino acids	Galas and Kaluzewska, 1992
*Streptomyces albidoflavus*	A stain was able to produce at least six proteases when it was grown in feather meal. A new enzyme SAKase with molecular weight of 18 kDa was identified	Bressollier et al., 1999
*Streptomyces gulbargensis*	A feather degrading strain produced keratinase that was stable at 45°C and pH 9.0 for 120 h	Syed et al., 2009
*Streptomyces pactum*	A 30 kDa protease was identified. This protease specifically cleaves substrates with Arg and Lys residues at the P1 site	Böckle et al., 1995
	Disulfide reduction was observed for the strain	Sangali and Brandelli, 2000
*Streptomyces fradiae*	A feather-degrading strain produced a 454 amino acids protease which can be overexpressed in *E. coli* and resulting product with a molecular weight of 25.6 kDa	Meng et al., 2007
*Streptomyces thermoviolaceus*	A strain was able to produce keratinase with a molecular weight of 40 kDa	Chitte et al., 1999
*Amycolatopsis*	The feather-degrading strain grown in feather medium could produce anti-fungus agents	Tamreihao et al., 2017
*Actinomadura*	A 29 kDa protease was produced by this strain grown in feather medium. The enzyme was stable at higher temperature and in detergent	Habbeche et al., 2014
*Scopulariopsis brevicaulis*	This strain was able to produce keratinase while this stain might be of secondary importance in the mineralization of keratinic substrates	Filipello Marchisio et al., 2000
*Trichophyton mentagrophytes*	This strain was able to produce keratinase using keratin-rich material. This stain was able to produce a 41 kDa keratinase whose PI was 3.9	Yu et al., 1968; Tsuboi et al., 1989
*Doratomyces microsporus*	A 30 kDa keratinase was purified and able to degrade different keratin materials	Gradišar et al., 2000
*Trichophyton rubrum*	This stain was able to produce keratinase using different substrates	Sharma et al., 2012
*Microsporum canis*	The keratinase activity plays a role in virulence of this fungus	Viani et al., 2001
*Candida albicans*	KPase was able to be produced and exhibited an optimal pH of 4.0	Negi et al., 1984
*Aspergillus flavus*	A 31 kDa keratinase was produced when this strain was cultured in a feather-containing medium	Kim, 2007
*Candida parapsilosis*	Treatment of the strain could enhance keratinase activity	Duarte et al., 2011
*Chrysosporium articulatum*	A strain was able to produce keratinase using feathers as sole carbon and nitrogen sources	Bohacz, 2016
*Aphanoascus fulvesnces*	This stain was isolated from soil and able to degrade feathers	Bohacz, 2016

## Keratinases

**FIGURE 2:**
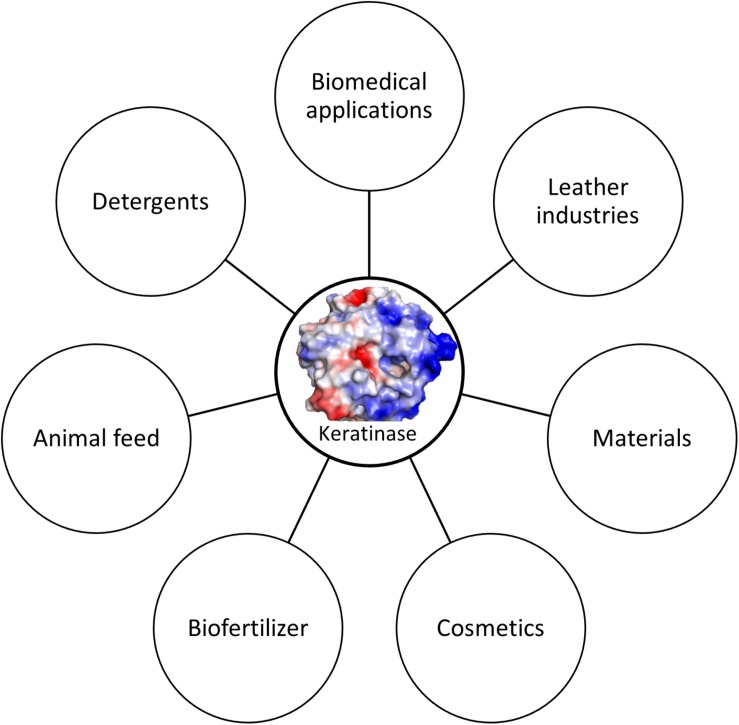
Keratinase applications. Keratinase can be used in different fields. The application can be enlarged when more stable enzymes are available.

The term keratinase (EC3.4.21/24/99.11) is referring to a class of proteases which possess keratinolytic activities (Lange et al., 2016). The identified keratinases are serine or metallo protease with the capability to degrade keratinous proteins (Gupta and Ramnani, 2006; Sahni et al., 2015). The mechanism of action of these enzymes is still not completely understood as these enzymes alone could not degrade native keratins (Gupta and Ramnani, 2006). The keratinases produced by various bacteria and fungi exhibit different characteristics such as amino acid sequence, molecular weight, optimal pH and temperature toward keratins from different origins (Brandelli, 2008). As keratinases are able to cleave insoluble and recalcitrant keratins derived from keratin-rich wastes such as feathers, hair and wool, they have great potential of industrial applications. Accumulated studies have shown that keratinase can be used in several fields (Figure 2) including animal feed (Grazziotin et al., 2006), fertilizers, leather industries, biomedical fields, detergents, cosmetics and materials (Yue et al., 2011; Paul et al., 2016; Su et al., 2019). Keratinase produced by *B. licheniformis* PWD-1 was found to be able to degrade prions which are infectious agents and resistant to proteolytic and mild protein-destructive processes (Van de Wiel et al., 2003). This enzyme can be used to remove prions present in animal feed. Keratinase will play important roles in agricultural and environmental chemistry due to its ability to degrade keratins from various sources (da Silva, 2018).

### Biochemical Analysis of Keratinases

Most researchers purified the enzyme from an organism and characterized the purified products using keratin-derived substrates such as azokeratin, keratin azure, human hair, cow horn, feather and keratin powder derived from different keratins (Gupta and Ramnani, 2006). The optimal enzymatic conditions including buffer pH, temperatures were obtained using these assays (Govarthanan et al., 2015). In addition, substrate specificity were analyzed by using substrates containing different amino acids (Brandelli et al., 2010). Keratinases from both bacteria and fungi exhibited activity under temperatures ranging from 28-90°C (Tamreihao et al., 2019) or even higher (Intagun and Kanoksilapatham, 2017). The enzymes could also sustain its activity at pH from 5 to 13 (Gupta and Ramnani, 2006; Brandelli et al., 2015). Studies also revealed that the enzymes from fungi, bacteria and other extremophiles exhibit higher optimal temperature, which gives rise to high efficiency in keratin degradation (Kanoksilapatham and Intagun, 2017). A heat-stable keratins from *Meiothermus taiwanensis* WR-220 was able to be over-expressed and purified by recombinant techniques (Wu et al., 2017). Biochemical and structural studies were able to be carried out to understand its mechanism of action. The presence of disulfide bonds in the protease contributes to the thermal stability (Wu et al., 2017). This information is very useful in enzyme engineering as some proteases do not contain cysteine residues. Introducing disulfide bonds will be a strategy to improve enzyme stability.

### Recombinant Keratinase

Recombinant DNA technology has been used to clone the genes of keratinases from bacteria and fungi and overexpressed them in bacteria such as *Escherichia coli* and other cells (Descamps et al., 2003; Khardenavis et al., 2009). Although it is not an economic way to use recombinant keratinases to degrade wastes such as feathers, introducing a tag to facilitate protein purification makes it straightforward for enzyme production with high purity and high yield. This method is also very useful for characterizing enzymes using biochemical, biophysical and structural methods (Fang et al., 2014). In addition, when this method is used in protein engineering, the activity and yield of the mutants are easily evaluated (Fang et al., 2019). With optimized conditions, recombinant production of keratinase is possible to be used for industrial applications.

### Structure and Substrate Reorganization Site of Keratinase

Structural studies have been carried out for several keratinases. One of the recent studies revealed the detailed structure of a keratinase (rMtaKer) from *M. taiwanensis* WR-220 (Wu et al., 2017), paving the way for understanding of mechanism of action of this class of enzymes. Intact rMtaKer consists of signal peptide, N-terminal pro-peptide (N-pro), and mature protease domain with a catalytic triad formed by Asp39, His72, and Ser224. The N-pro region was cleaved when rMtaKer was overexpressed in *E. coli*, resulting in the protease domain only. The purified rMtaKer exhibited active protease activity against several substrates such as feathers, milk, casein and elastin. The 1.5 Å crystal structure of the protease shows that keratinase is composed of seven-stranded parallel β-sheets with six α-helices and four β-sheets flanking around. The four β-sheets are made of two anti-parallel strands and possess the conserved catalytic triad formed by Asp39, His72, and Ser224.

**FIGURE 3:**
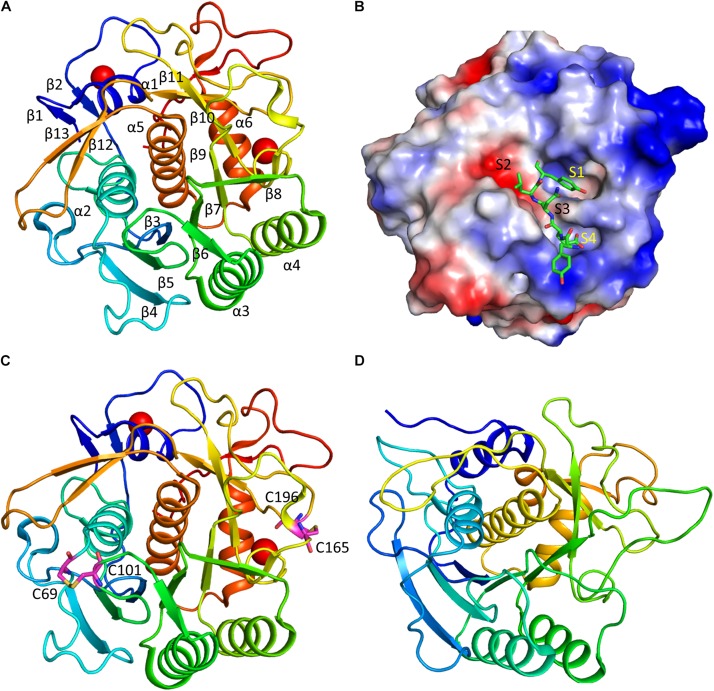
Structure and substrate binding of keratinases. **(A)** Crystal structure of rMtaKer. The structure of rMtaKer (PDB id 5WSL) is shown. The metal ions are shown as spheres. **(B)** Surface charge representation of rMtaKer. The substrate binding to the active site are shown as sticks. **(C)** The residues critical for disulfide bond formation are shown. **(D)** Crystals structure of other keratinase. The crystal structure of a bacterial keratinase (PDB id 1CSE) is shown. No cysteine residue is present in this enzyme.

The keratinase contains two calcium ions that are important for stabilizing the structures. The presence of the metal ions in keratinase explains the fact that many ions are important for affecting enzymatic activity. The first Ca^2+^ stabilizes the surface loop between α1 and β2, coordinated by oxygen atoms from Asp11, Asp14, Gln15, Ser21, and Thr23. The second Ca^2+^ exhibits close contacts with oxygen atoms of Val172, Gly175, Thr177, and two water molecules. These two metal ions are also present in other members of the subtilisin superfamily. Two disulfide bonds formed by residues Cys69-Cys101, and Cys165-Cys196 (Figure 3). As the disulfide bond is able to stabilize protein structure, the optimal temperature for rMtaKer is 65°C. The presence of the disulfide bonds might be one of the reasons for the thermal stability. Introducing disulfide bonds to other keratinase in protein engineering will be a strategy to improve protease thermal stability.

The substrate specificity of keratinases is still not well known. A study showed that *Nesterenkonia* sp. AL20 was able to produce alkaline protease using chicken feather as the nutrient source. The protease was able to cleave tetra-peptide substrates with hydrophobic residues at the P1 site (Bakhtiar et al., 2005). In the structure of rMtaKer, self-cleavage was observed. Although rMtaKer is monomeric in solution, protein form oligomers in the crystals, which is due to crystal packing. The residues including Tyr278-Glu279-Asn280-Leu281-Tyr282 occupy the protease active site from the neighboring monomer (Figure 3). This structure reveals the residues that are critical for substrate binding. The protease cleavage sequence from P1 to P4 obtained in this study can be used for determining the optimal protease cleavage site.

### Constructing More Active and Stable Enzyme

Keratinases from microorganisms are present in a pre-pro-form in which an inhibitory region suppresses the protease activity (Liu et al., 2014). This inhibitor region acts as an intermolecular chaperone critical for the folding of the mature protease domain by assisting its folding, affecting the structure of the protease domain and temporarily binding to the protease domain before activation through *cis* or *trans* modes (Liu et al., 2014). Mutations in the pro-peptide region can affect the folding rate of the protein, which leads to high enzyme production, conformational change of the mature enzyme and acceleration of enzyme maturation. Such studies have been carried out on proteases from several species (Takagi et al., 2001; Fang et al., 2010; Uehara et al., 2013; Su et al., 2019). Computational aided site-directed mutation was carried out for residues at different sites. Mutation of residues (Y94F) was able to improve substrate specificity. Some mutations such as A218S and A218G were able to improve the thermal stability of the enzymes. The mutants carrying mutations at this site exhibited highest activity at 70°C, which will broaden the application of keratinase (Fang et al., 2017). These mutagenesis studies are also helpful for understanding the dynamics and regulation of keratinase (Liu et al., 2013).

### Mechanism of Keratin Degradation by Microorganisms

Structural studies on keratinases and feather degradation *in vitro* suggested that one keratinase is not enough to degrade keratin as keratinases do not have the activity to break the disulfide bonds. Several mechanisms have been proposed and it has been recognized that two steps may be involved in the keratinolytic process- sulfitolysis and proteolysis (Korniłłowicz-Kowalska and Bohacz, 2011). Sulfitolysis is required to cleave disulfide bonds and proteolysis is to cleave the protein (Yamamura et al., 2002b). Removal of the disulfide bonds by enzymes such as sulfide reductases or reducing agents such as sulfite secreted by the strain is critical for conformational changes of keratins, which makes more sites available for keratinase degradation (Lange et al., 2016). A study has shown that the crude enzyme exhibited higher keratin degrading rate than the purified enzyme. At least two enzymes are required for keratin degradation (Yamamura et al., 2002a). One enzyme is responsible for producing reduced keratin with cysteine residues in the reduced form so that its cleavage sites were exposed to protease. The following steps are also proposed in keratin degradation, namely sulfitolysis, proteolysis, and deamination (Yu et al., 1968). Bacteria and fungi exhibit different mechanisms for keratin degradation while their keratinases are able to cleave the polyproteins. In addition to sulfitolysis and proteolysis, mechanical destruction plays important roles in keratin degradation by fungi (Korniłłowicz-Kowalska and Bohacz, 2011). Despite such progress in understanding protease mechanism of action, more studies have to be carried out to identify the enzymes critical for keratin degradation.

## Questions Need to Be Solved

Studies on keratinases produced by both bacteria and fungi have been carried out by researchers (Table 1) and reviewed (Gupta and Ramnani, 2006; Brandelli, 2008; Brandelli et al., 2010; Korniłłowicz-Kowalska and Bohacz, 2011; Gupta et al., 2013). Different keratinases have been purified and characterized from various stains (Table 1). Accumulated studies suggest that other enzymes such as disulfide reductase that can break disulfide bonds are important for keratinase degradation. Studies have shown that the whole-cell product has a higher keratin-degrading efficiency than the purified keratinase does. As proposed in a recent review, enzymatic degradation of keratins might be similar to decomposition of cellulose in which several enzymes are required in the process. Further studies identifying new components important for keratins degradation are important for understanding the mechanism of action of keratinases. It has been noted that keratins do not accumulated in the nature, suggesting that they might be degraded through the cooperation of microorganisms such as bacteria and fungi in the environment. Extensive studies have been working on screening strains that are able to degrade feathers or keratins while most studies were focused on a single strain in keratin treatment.

## Perspective

Feathers from poultry industries can be a valuable resource instead of a waste or a threat to the environment. Based on accumulated studies, there is no doubt that feathers can be used in diverse fields. Microbial degradation of feathers is a feasible and economic way to make full use of the waste. Converting feathers into biofertilizer and animal feeds is easily applicable to various poultry industries. Despite the efforts spent in this direction, some works are still necessary to be carried out to build up more efficient systems that can be utilized in feather treatment.

### Strategies in Strain Screening

Feather degrading pathogenic bacteria and fungi are useful for understanding the mechanism of action of keratinases, but these strains must not be used in feather treatment due to their pathogenic effect. These strains can be easily isolated as they exist widely in nature. Careful strain identification is needed in strain isolation and identification steps. In addition, the antifungal or antibacterial activity can be used as a parameter in the screening step. A study showed that feather-degrading *B. subtilis* S8 exhibited antifungal activity (Go et al., 2015) and other feather-degrading strains also exhibited antibiotic activity. When a strain is isolated and characterized, whole-cell mutagenesis is an efficient strategy to improve its feather degradation capabilities. All these studies are important to obtain potent microorganisms for feather degradation. It has been noted the fermentation conditions are critical to make the microorganisms to exhibit high yield of the target enzymes (Tatineni et al., 2007).

### Microbial Consortium

Combination of several stains might be a strategy to degrade feathers efficiently. Indeed, a research team has screened a microbial consortium KMCG6 which is composed of Bacteroidetes and Proteobacteria. This consortium exhibited a high degradation level (Kang et al., 2018). Similar strategy can be utilized in screening suitable stains that are able to degrade feathers efficiently. It has been noted that microbial consortium or mixed microorganisms have different effects from a single microorganism in microbial degradation field. Careful experimental design is required in the steps of enrichment and screening. It might be possible that combining isolated stains to construct a microbial consortium is easy to obtain reproductive results in later applications.

### Keratinase Identification

Many microorganisms are able to produce keratinases. With the development of genome sequencing techniques and accumulation of genome sequences in nucleotide databases, analyzing the information from the genome can provide a fast way to identify new enzymes that are critical for keratin degradation. Using this approach, the enzymes critical for feather degradation were analyzed for anaerobic bacterium *Serratia marcescens* EGD-HP20 (Fuke et al., 2018). This approach might not be sufficient to identify enzymes that are required for feather degradation as the enzymes secreted into the medium are affected by cultural conditions (Rajput and Gupta, 2013). In addition to optimizing fermentation conditions to achieve high feather-degrading efficiencies, identification of secreted enzymes critical for keratin degradation should be carried out using sensitive methods such as mass spectrometry (MS). Combination of DNA analysis with MS identification on secreted proteins will provide a reliable and fast way to identify necessary components in keratin degradation.

### Keratinase Characterization

Several types of substrates have been used in enzymatic characterization while using keratin-driven substrates which may not be sensitive enough to characterize different enzymes due to the solubility. Developing an efficient enzymatic assay in which peptidic substrate is used will be useful for characterizing and comparing keratinases from different microorganisms. Based on recent crystal structure of a keratinase, the five-residue peptide Tyr278-Glu279-Asn280-Leu281-Tyr282 can serve as a keratinase substrate in protease activity assay. It has been noted that the optimization of the peptide sequence might be required for obtaining more sensitive substrate.

### Enzyme Engineering

Protein engineering of keratinase has been successful in creating more stable enzymes under high temperatures and alkaline conditions. Bioinformation-guided point mutation is an efficient way to alter enzymatic characteristics (Fang et al., 2016, 2019). The structure of a thermal stable keratinase can be used to guide enzyme engineering. For example, disulfide bonds can be introduced into a keratinase to increase the enzyme stability (Wu et al., 2017). Replacing Ca^2+^-binding residues with other amino acids could possibly abolish mental binding activity of keratinases, which could reduce the reliance of metal ions in enzyme catalysis. Accumulated studies prove that it is feasible to construct more active and stable enzymes that are suitable for industrial applications.

### Mixed Enzymes in Feather Degradation

Keratinases have great potential to be used in several fields. Accumulated studies have suggested that several enzymes are essential for keratin degradation. Mixed enzymes might be required for developing keratinase products which can have higher keratin degrading activity. More studies on enzymes such as disulfide reductase will be useful for designing keratinase products. In addition, adding some chemicals that can facilitate breakage of the disulfide bonds is also useful for increasing keratinase catalytic efficiency.

In summary, with the increase of global population, the requirement for poultry products such as broiler is growing annually. The by-product, feather is also an important source that can be converted from potential wastes to value added products. Microbial processing of feather is therefore a promising technique to generate versatile products such as biofertilizer, animal feed and keratinases. More studies are still required to understand mechanism action of feather degradation and set up an economic strategy for feather processing in large scales.

## Author Contributions

QL drafted and revised the manuscript.

## Conflict of Interest

The author declares that the research was conducted in the absence of any commercial or financial relationships that could be construed as a potential conflict of interest.
